# Antibiotic prescribing in patients with acute rhinosinusitis is not in agreement with European recommendations

**DOI:** 10.3109/02813432.2013.788270

**Published:** 2013-06

**Authors:** Lars Christian Jørgensen, Sarah Friis Christensen, Gloria Cordoba Currea, Carl Llor, Lars Bjerrum

**Affiliations:** ^1^Section of General Practice and Research Unit for General Practice, Department of Public Health, University of Copenhagen, Copenhagen, Denmark; ^2^Spanish Society of Family Medicine, Primary Healthcare Centre Jaume I, University Rovira i Virgili, Tarragona, Spain

**Keywords:** Antibiotics, Denmark, diagnosis, general practice, inappropriate prescribing, recommendations, sinusitis

## Abstract

**Objective:**

To assess the potential overprescribing in patients with acute rhinosinusitis across six countries with different antibiotic prescribing rates and different prevalence of antibiotic resistance.

**Design, setting and subjects:**

A cross-sectional study including GPs from two Nordic countries, two Baltic countries and two Hispano-American countries registered patients with respiratory tract infections (RTIs) during three weeks in January 2008 as part of the EU-funded project “Health Alliance for Prudent Prescribing, Yield And Use of antimicrobial Drugs In the Treatment of respiratory tract infections” (HAPPY AUDIT).

**Main outcome measures:**

Use of antibiotics for acute rhinosinusitis based on the recommendations in the European Position Paper on Rhinosinusitis and Nasal Polyps 2007 (EP3OS).

**Results:**

In total, 618 participating GPs registered 33 273 patients with RTI of whom 1150 (3.46%) were considered to have acute rhinosinusitis. Over 50% of the patients with acute rhinosinusitis had symptoms for < 5 days and 81% were prescribed antibiotics. In total, 68% of the patients included were not prescribed antibiotics according to guidelines; 45% had symptoms < 5 days or no fever (possible overprescribing) and 23% had symptoms < 5 days and no fever (probable overprescribing).

**Conclusion:**

A considerable number of patients with symptoms of acute rhinosinusitis were not managed according to European recommendations (EP3OS guidelines). To prevent overprescribing, efforts should be made to implement the recommendations in daily practice.

- According to European recommendations, antibiotics should only be prescribed in patients with increasing or persisting severe symptoms of rhinosinusitis.
- The majority of patients classified with the diagnosis rhinosinusitis do not meet the criteria for the diagnosis according to European recommendations.
- About half of the patients with rhinosinusitis are exposed to possible antibiotic overprescribing and approximately 25% to probable overprescribing.
- To prevent overprescribing, efforts should be made to implement the European recommendations concerning acute rhinosinusitis in daily practice.

## Introduction

Acute rhinosinusitis is one of the most common reasons for visiting a doctor, and the illness imposes major economic costs on society in terms of direct costs as well as decreased productivity. Today, acute rhinosinusitis is the fifth most common diagnosis for which an antibiotic is prescribed in primary care settings in the Nordic countries [1], accounting for up to 21% of all antibiotic prescriptions. Despite the fact that only a small percentage of viral upper respiratory tract infections (URTIs) are complicated by bacterial infection, more than 80% of patients with symptoms of rhinosinusitis are prescribed antibiotics [2]. Several studies have shown that antibiotics provide few if any benefits in patients that are classified clinically with the diagnosis acute rhinosinusitis [3,4].

Acute bacterial rhinosinusitis is most often preceded by a viral upper respiratory tract infection, and develops as a secondary infection due to impaired mucus clearance. No simple and accurate office-based test for acute bacterial rhinosinusitis exists and general practitioners (GPs) mostly rely on clinical findings when stating the diagnosis. Imaging leads to a high number of false positive and negative results and the use of nasal endoscopy is not feasible in general practice. Differentiating the bacterial or viral origin of rhinosinusitis is very challenging, which makes it difficult to decide whether or not to prescribe antibiotics [5]. Undoubtedly, this uncertainty leads to overprescribing of antibiotics, which is considered to be an important reason for development of bacterial resistance to antibiotics [6–8].

The European Position Paper on Rhinosinusitis and Nasal Polyps 2007 (EP3OS) provide evidence-based guidelines on the diagnosis and treatment of rhinosinusitis in general practice [9]. The EP3OS guidelines recommend how to identify and manage acute bacterial rhinosinusitis, based on symptoms alone. In brief, antibiotics should only be prescribed in patients with severe symptoms including fever (> 38°C) and severe pain (visual analogue score > 7), and the symptoms should increase after five days or persist for more than 10 days. A shorter duration of symptoms (< 10 days) is an indicator of a viral infection and such patients should not be treated with antibiotics.

Due to the observed high prescribing rate of antibiotics in patients with rhinosinusitis we hypothesized that patients were not managed in accordance with the EP3OS recommendations. Particularly, we suspected an overprescribing of antibiotics because the criteria concerning duration of symptoms (> 5 days) and fever were not met.

The aim of this study was to assess the potential overprescribing of antibiotics in patients with acute rhinosinusitis across six countries with different antibiotic prescribing rates and different prevalence of antibiotic resistance.

## Material and methods

Data for this cross-sectional study are based on the EU-funded project “Health Alliance for Prudent Prescribing, Yield And Use of antimicrobial Drugs In the Treatment of respiratory tract infections” (HAPPY AUDIT). Detailed information regarding this project can be found in the HAPPY AUDIT study protocol [10].

In brief, data from general practice were collected by auditing GPs in six countries: two Nordic countries (Denmark and Sweden), two Baltic countries (Lithuania and Russia), and two Hispano-American countries (Spain and Argentina). Using a prospective self-registration methodology, based on a chart filled in by the GP during consultation, patients with respiratory tract infections (RTIs) were registered during a three-week period in the winter months of 2008. For each patient the GP registered symptoms and signs, presumed diagnosis, and treatment.

We assessed the potential overprescribing in patients with suspected acute rhinosinusitis using the EP3OS recommendations as benchmark criteria for the diagnosis and treatment. We considered “possible” antibiotic overprescribing if patients were treated with antibiotics in spite of symptom duration < 5 days *or* no fever. We considered “probable” antibiotic overprescribing if patients were treated with antibiotics in spite of symptom duration < 5 days *and* no fever.

The data were analysed in Statistical Analysis Software (SAS) version 9.2. and Microsoft Office Excel 2007.

## Results

In total, 618 GPs participated in the study and they registered a total of 33 273 patients with RTIs. Altogether, 1150 (3.5%) of patients with URTI were considered to have acute rhinosinusitis, but the proportion varied between 2.4% (Argentina) and 7% (Denmark).

Table I shows characteristics of the patients included. The majority of the patients were women and the median age was 35 years, ranging from 19 years (Lithuania) to 45 years (Sweden). About one-third had fever at first presentation.

**Table I.** Characteristics of patients included.

Country	No. of patients (%)	No. of patients with RTI (% rhinosinusitis)	No. of women (%)	Median age (iqr)	No. of patients with fever (%)	Median no. of days with symptoms (iqr)	No. of patients with < 5 days of symptoms (%)	No. of patients prescribed antibiotics (%)
Argentina	105 (9.1)	4374 (2.4)	70 (66.7)	25 (14–35)	50 (47.6)	4 (2–5)	80 (77.7)	90 (85.7)
Denmark	272 (23.7)	3904 (7.0)	187 (68.8)	41 (32–55)	83 (30.5)	7 (4–12)	104 (39.3)	203 (74.6)
Lithuania	74 (6.4)	2706 (2.7)	29 (39.2)	19 (12–35)	26 (35.1)	5 (3–7)	43 (60.6)	69 (93.2)
Russia	134 (11.7)	3685 (3.6)	77 (57.5)	24 (6–42)	49 (36.6)	3 (2–5)	108 (83.1)	81 (60.5)
Spain	445 (38.7)	16751(2.7)	277 (62.3)	36 (28–48)	150 (33.7)	5 (3–9)	227 (52.1)	388 (87.2)
Sweden	120 (10.4)	1853 (6.5)	87 (72.5)	45 (33–57)	34 (28.3)	10 (7–14)	18 (15.7)	105 (87.5)
Total	1150 (100)	33273 (3.5)	727 (63.2)	35 (25–48)	392 (34.1)	5 (3–10)	580 (51.8)	936 (81.4)

The median number of days with symptoms before first consultation was five days, but varied from three days (Russia) to 10 days (Sweden). More than half of all patients had symptoms for less than five days, but this proportion varied considerably, ranging from 16% (Sweden) to 83% (Russia). About 80% of all patients were prescribed antibiotics. The highest prescribing rate was found in Lithuania (93%).

**Figure 1.**
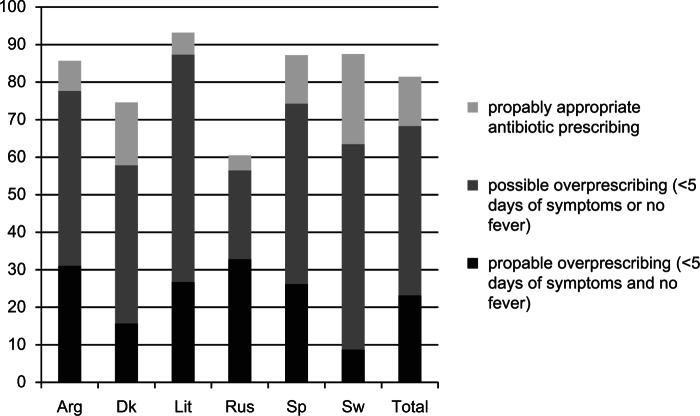
Antibiotic prescribing rates (%) for patients with acute rhinosinusits in six countries.

Figure 1 shows the antibiotic prescribing pattern for the countries included. The lower part of the columns corresponds to the percentage of patients treated with antibiotics, but who had symptom duration < 5 days and no fever (probable overprescribing). The middle part corresponds to the patients treated who either had symptom duration < 5 days or no fever (possible overprescribing). The upper part corresponds to the percentage of patients prescribed antibiotics with symptom duration of > 5 days and fever (probably appropriate prescribing).

The rates of potential overprescribing varied significantly between the countries. In total, 68% of patients were not prescribed antibiotics according to guidelines; 45% were exposed to possible overprescribing and 23% were exposed to probable overprescribing.

## Discussion

We found that more than 80% of patients with acute rhinosinusitis were prescribed antibiotics – a high number, considering that only a small percentage of viral URTIs are complicated by bacterial infection.

According to the EP3OS guidelines, only severe cases of acute bacterial rhinosinusitis should be treated with antibiotics. Fever should be present and symptoms should have a duration of at least five days.

However, in our study a considerable number of the patients treated with antibiotics did not meet the EP3OS criteria. Thus, they were exposed to potential antibiotic overprescribing. More than two-thirds of patients were not prescribed antibiotics according to guidelines; nearly half of patients were exposed to possible overprescribing (duration of symptoms < 5 days or no fever) and approximately 25% to probable overprescribing (duration of symptoms < 5 days and no fever).

Our results also showed that the rates of potential overprescribing varied significantly between countries, ranging from 56% in Russia to 87% in Lithuania. The rates of probable overprescribing were lowest in the Nordic countries. These variations suggest different management of rhinosinusitis and different attitudes between countries towards the use of antibiotics for acute rhinosinusitis. This may be due to discrepancies in national recommendations, different health care systems, different treatment traditions, different culture, different patient expectations, or different impact of marketing by pharmacies and pharmaceutical companies. The large differences in median age of patients between countries indicate that there are considerable variations in ways to establish the diagnosis of rhinosinusitis.

We may have underestimated the rate of antibiotic overprescribing in patients with acute rhinosinusitis. We only looked at fever and the number of days with symptoms at first contact. Some of the patients with symptom duration > 5 days may, however, not have met the EP3OS criteria of increasing symptoms.

When using adherence to guidelines as an estimate of the amount of overprescribing, it is paramount to be critical towards the guidelines. Developed by a group of both specialists and primary care physicians, the EP3OS guidelines include a comprehensive review of diagnostic methods and treatments for acute rhinosinusitis. That antibiotics should only be prescribed in patients with symptom duration > 5 days (or in severe cases) was based on the highest level of evidence and therefore strongly recommended [9].

Recommendations on how to establish the diagnosis of rhinosinusitis were not graded by evidence, and the predictive value of the symptoms for acute bacterial rhinosinusitis is not presented in the guidelines. Furthermore, it is unclear to what extent the recommendations for primary care were based on studies performed in primary care.

In the EP3OS guidelines, C-reactive protein measurements were briefly mentioned as helpful to exclude suspicion of a bacterial infection. In the updated EPOS guidelines from 2012, however, the recommendations have been revised, and severe acute rhinosinusitis is now defined as the presence of at least three of the following; discoloured discharge, severe local pain, fever, double sickening, or elevated CRP/ESR [11]. Several studies have shown that the use of point-of-care tests may lead to lower antibiotic prescribing for acute rhinosinusitis [12,13]. In our study, CRP measurements were virtually only used in the Nordic countries, which may be part of the explanation why the rates of probable overprescribing were lowest in these countries.

GPs participated on a voluntary basis and their prescribing habits may therefore not represent the average use of antibiotics in their respective countries [14]. GPs willing to register their antibiotic prescribing and to dedicate sufficient time to complete patient reports without economic incentives may have been more interested in quality development and research than GPs in general. Moreover, performing registration of antibiotic prescribing may in itself influence the prescribing habits. However, studies have shown that the reliability of the methodology is high and findings are correlated with the real prescribing rate in practice [15].

In general practice, the diagnostic procedure and the decision to prescribe are intricately linked [16,17]. The GP may decide whether or not to prescribe an antibiotic at the same time as, or even before, classifying a specific diagnosis to fit the decision on treatment. Previous studies have demonstrated that many factors can influence antibiotic prescribing in general practice; e.g. GPs’ consultation rates [18], patients’ expectations regarding antibiotics, overestimation of patients’ expectations by the general practitioner, and public knowledge about RTIs and antibiotics [19–21].

A Finnish cross-sectional survey found that duration of symptoms was a weak predictor of antibiotic prescribing and that patients with acute sinusitis were prescribed antibiotics two to five times more often than the true disease incidence would suggest [22]. This indicates that there is a diagnostic misclassification bias in general practice leading to an over-diagnosing of acute rhinosinusitis.

Our results are in accordance with previous studies showing prescribing rates for rhinosinusitis as high as 92% in the United Kingdom [23], 98% in the United States [24], 97% in Sweden [25], 83% in Finland [22], 60% in Holland [26], and 70% in Denmark [15]. Reflecting the fact that the aetiology is almost always viral, these high figures suggest that the overprescribing for acute rhinosinusitis is a global problem.

A Dutch study from 2005 [19] including 146 GPs and 581 patients with acute rhinosinusitis assessed inappropriate use of antibiotics when using Dutch national recommendations on antibiotic prescribing as benchmark criteria. They found that almost half of the patients had symptoms for > 2 weeks prior to consultation and 22% were exposed to antibiotic overprescribing. Surprisingly, they also found 8% of underprescribing.

Our results indicate that a considerable number of patients with symptoms of acute rhinosinusitis are not managed according to the internationally agreed recommendations described in the EP3OS guidelines. Studies have shown that implementation of guidelines can be challenging. It has been debated whether a single or a multifaceted intervention produces better results in guideline implementation and the question remains unanswered [27]. The HAPPY AUDIT project showed that a multifaceted intervention programme that included training courses, clinical guidelines, posters for waiting rooms, patient brochures, and access to point-of-care tests may lead to a marked reduction in antibiotic prescribing in patients with RTIs [28]. To prevent overprescribing, such efforts should be made to implement the new EPOS recommendations in daily practice. In particular, it is paramount to evaluate the severity of the symptoms and take the duration into consideration before making any decision on antibiotic prescribing.
